# Introduction to the themed issue on the design, development, evaluation, and dissemination of team science interventions in clinical and translational research

**DOI:** 10.1017/cts.2021.870

**Published:** 2021-12-02

**Authors:** Betsy Rolland, Jennifer E. Cross, Sarah D. Hohl, LaKaija J. Johnson, Kevin Wooten, Allan R. Brasier

**Affiliations:** 1 Institute for Clinical and Translational Research, School of Medicine and Public Health, University of Wisconsin-Madison, Madison, WI, USA; 2 Carbone Cancer Center, School of Medicine and Public Health, University of Wisconsin-Madison, Madison, WI, USA; 3 Colorado Clinical and Translational Sciences Institute, Institute for Research in the Social Sciences, Sociology, Colorado State University, Fort Collins, CO, USA; 4 Office of the President, University of Houston Clear Lake, Houston, TX, USA; 5 Institute for Translational Science, University of Texas Medical Branch, Galveston, TX, USA; 6 Internal Medicine, School of Medicine and Public Health, University of Wisconsin-Madison, Madison, WI, USA

**Keywords:** Team science, translational teams, interventions

## Introduction

From the COVID-19 pandemic to the future of population health in a changing climate [1], it is clear that translational science teams are critical to solving complex, multidimensional problems that impact population health. In the past two years, we have witnessed rapid deployment of teams across the biomedical research enterprise focused on developing treatments and prevention strategies for a previously little understood disease and collaborating with communities to mitigate health inequities exacerbated by the pandemic [2]. Despite tangible success, we have also seen that such translational teams are often formed, managed, and led without the benefit of evidence-based guidance to help them work together efficiently and effectively. This problem is emblematic of translational teams more broadly. The field of the Science of Team Science (SciTS) has identified several characteristics and practices of effective teams that are associated with improved team functioning, such as deep knowledge integration, skilled leadership, and a climate of trusting relationships [3]. The ways in which a specific Clinical and Translational Research (CTR) team can develop those characteristics or precisely which activities will help a particular team achieve success, however, have been less clear. The result of this *research-practice gap* is that CTR teams who want to improve may struggle to effectively identify, obtain, and implement the resources needed to do so.

The focus of this themed issue is on the design, development, evaluation, and dissemination of Team Science interventions in CTR. The impetus for this issue was the relative dearth of evidence-based interventions to support team effectiveness, which can leave translational scientists to fend for themselves in establishing effective teams. It can be a daunting task for a busy translational scientist to identify the team processes they are interested in improving, navigate the SciTS literature, evaluate proposed interventions (few of which themselves evaluate either efficacy or effectiveness), implement those interventions, and then assess the intervention’s impact. The CTR community requires evidence-based, accessible, active, actionable interventions with defined outcomes that are easy for CTR network or initiative leaders to find, access, implement, and assess. Furthermore, CTR teams require different kinds of interventions that act upon the multiple levels that impact team-based research: the individual scientist, the team, the institution, and the overarching system of scientific research. The papers included in this themed issue provide practical guidance for designing, implementing, and evaluating such interventions, as well as proposed frameworks for developing this aspect of SciTS research.

## Challenges of Intervention Development

The CTR community is familiar with the challenges of developing interventions to impact human health; many of those challenges hold for developing interventions to improve team effectiveness. First, we need improved definition of our audiences and their challenges. Translational research initiatives, by definition, are made up of diverse teams of scientific experts, community partners, policy makers, research staff, funding agencies, and/or industry partners [4]. Developing interventions to meet the needs of those individuals and teams requires an understanding of how these different players contribute to translational research, how they interact with one another, and how their needs and priorities impact their ability to contribute as a true part of a team. Second, we need improved methods for developing interventions to ensure scientific rigor and generalizability of our interventions, which will allow us to develop and adapt interventions more rapidly. Here, again, we can learn from our counterparts developing health interventions, as well as those developing technologies such as apps and games, leaning on the methods they have developed to provide situational training and skill enrichment. Specifically, we can integrate the approaches, methods, and techniques of our Dissemination & Implementation (D&I) counterparts in the pragmatic testing and evaluation strategies used to show efficacy and then effectiveness. In addition, because many team science interventions are tested on a small scale, they may not be feasible for broader use or with large teams. Leveraging D&I approaches for spread and scale-up, integrating approaches into the organizational culture and practices, engaging diverse translational teams in design, and involving the broader team science community in testing the interventions will improve the rigor and ease of adoption of interventions. Finally, we must develop common approaches to evaluating the effectiveness of team-based interventions after they have been released into the wild. The common data element approach used for evaluation of CTSA workforce development programs could be adapted to evaluate the impact of team science interventions across organizations [5].

## Goals of This Themed Issue

The goals of this Themed Issue are to raise awareness of the “research-practice” gap, to formalize thinking on the process of developing evidence-based interventions, identify emerging themes in team science, and identify areas for future research. A challenge encountered in this issue was settling on a definition of what constituted a team science *intervention* for translational science teams. We sought to be as inclusive as possible to accommodate the relative newness of this question for the SciTS field, while being specific enough to move the field toward a common understanding. We propose the following definitions:

We define a translational team science *strategy* as a program, policy, or practice designed to improve translational team processes, outputs, outcomes, and/or translational science benefits. We broadly define a translational team science *intervention* as one strategy or a set of strategies that have been systematically implemented with a team and evaluated, with demonstrated impact. *Evidence-based interventions*, then, represent strategies that have been tested, with rigorous evaluation of their impact on team process in real-world settings [6].

**Fig. 1.**
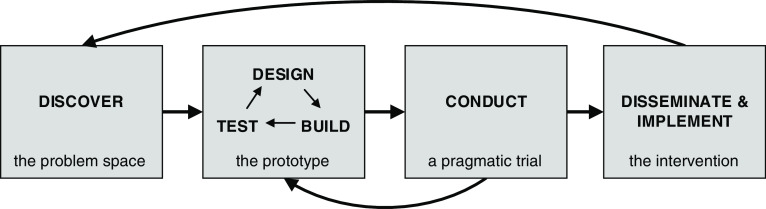
Wisconsin Interventions for Team Science framework: a four-phase approach to team science intervention development [6].

Translating best practices from human-centered design and implementation science provides a general framework for discovering, testing, and disseminating these evidence-based interventions, as exemplified in Fig. 1, below, from Rolland et al. [6].

## Emerging Themes and Future Research Directions

The guest editors for this issue selected submissions that represent a deliberate intention of impacting team effectiveness and improved scientific impact. The following themes emerged from this set of manuscripts:

- Translational teams have team science needs that are specific to the characteristics of CTR, including needing techniques to deal with the challenges of increased interdisciplinarity, the engagement of community partners and other lay stakeholders, and the focus on the development of some sort of product (e.g., intervention, drug, device), which often intensifies intellectual property concerns. Furthermore, CTR teams have different needs at different points in that lifecycle, requiring a variety of interventions and ways to understand which interventions work in different contexts [7–9]. While most of the interventions described in this issue target early team stages, there is clearly a need for interventions throughout the lifecycle of a translational team. Our goal should be to make engagement with team science interventions a *de facto* part of leading and managing a team. To accomplish this, we will need policy support to encourage or require the integration of evidence-based methods of team development.
- Team science interventions can and should occur at multiple levels, from workshops for individuals to whole-team activities to improve teamwork to institution-wide approaches to facilitating team science [10–12]. These interventions should integrate with the increasing number of training opportunities being offered by CTSA hubs, creating “cradle-to-grave” support for teams and the individuals who comprise them [10,12–14]. Teams may need some or all of these interventions. Making them available “on demand” as teams encounter developmental challenges will be key to their adoption and effectiveness. Effective and systematic team assessments to help teams know which interventions to incorporate and when would be beneficial [9].
- The interventions described in this issue had varying outcomes as their goals, ranging from participant satisfaction with a workshop to improved competency of individuals in team science skills as measured by validated surveys. However, the ultimate objective of the team science interventions was often less clear; this is an area for growth in the SciTS field [6,7,15]. Is it the improvement of team effectiveness? Greater team satisfaction? More high-impact science? More efficient adoption into health care? What can an individual, a team, or an institution expect to gain from participating in a given intervention and how will that gain be measured in a rigorous and reproducible way?
- The methods and approaches for the development of team-based interventions are still in their infancy. Several papers in this collection are focused specifically on defining the process itself [6,15,16], with others describing the process they used in intervention development in detail [8,13]. The SciTS field needs more rigorous methods for every stage of the intervention development process, but we do not need to start from scratch. As proposed in this issue, the fields of human-centered design, implementation science, and health intervention development can be leveraged to develop methods specifically geared toward meeting the needs of translational teams [14–17]. Special attention is needed for the evaluation and dissemination/implementation stages of intervention development if we are to make claims of efficacy and effectiveness for our interventions. Teams must be able to trust that, if they invest time in an intervention, they are likely to improve their ability to collaborate and deliver on their team’s objectives. Team science intervention developers at CTSAs should consider engaging their Dissemination & Implementation teams to help design for dissemination and build interventions that can be disseminated easily.
- Many, if not most, of the interventions described here require facilitation by team science experts, raising issues of scalability [6,8,14]. The SciTS field remains relatively small, in part because of a lack of explicit training or career paths and in part because of the challenges of funding such positions. For team science interventions to become an expected part of collaborative science projects, team science facilitators must become an expected part of a science team and/or a central support resource within institutions [18–20]. If we can evolve to the point where teams using team science expertise and interventions are doing substantially, measurably more high-impact science than teams that are not, integration of scientific facilitation becomes an issue of stewardship and ethical use of resources. This acceptance of team science as a *method* may lead to its integration into the design of any collaborative research project, akin to the inclusion of a biostatistician or clinical trialist.

## Conclusion

The Science of Team Science field has tremendous potential for developing and delivering team science interventions that increase team effectiveness, which can result in high-impact science, meaningful health care interventions, and population health improvements. Both SciTS and CTR will benefit greatly from the development and application of rigorous methods of intervention development that result in evidence-based interventions that are accessible, active, actionable, and replicable. Adopting common assessments will enable the understanding of the impact of specific interventions in diverse teams and institutions. These interventions, aimed at individuals, teams, and institutions, at all points along the lifespan of a translational team, can make team-based research more efficacious, encouraging more collaborations to tackle the most pressing scientific problems of our time.
